# Transactivation in *Drosophila* of Human Enhancers by Human Transcription Factors Involved in Congenital Heart Diseases

**DOI:** 10.1002/dvdy.22763

**Published:** 2011-10-10

**Authors:** Vincenzo Amodio, Maria Florencia Tevy, Concetta Traina, Tushar Kanti Ghosh, Maria Capovilla

**Affiliations:** 1 Dulbecco Telethon Institute, Department of Biology and Evolution, University of FerraraFerrara, Italy; 2 School of Biology, Institute of Genetics, Queen's Medical CentreNottingham, United Kingdom

**Keywords:** *Drosophila*, heart, transcription factor, Congenital Heart Disease, UAS/GAL4

## Abstract

Background: The human transcription factors (TFs) GATA4, NKX2.5 and TBX5 form part of the core network necessary to build a human heart and are involved in Congenital Heart Diseases (CHDs). The human *natriuretic peptide precursor A (NPPA)* and *α-myosin heavy chain 6 (MYH6)* genes are downstream effectors involved in cardiogenesis that have been demonstrated to be *in vitro* targets of such TFs. Results: To study the interactions between these human TFs and their target enhancers *in vivo*, we overexpressed them in the whole *Drosophila* cardiac tube using the UAS/GAL4 system. We observed that all three TFs up-regulate their natural target enhancers in *Drosophila* and cause developmental defects when overexpressed in eyes and wings. Conclusions: A strong potential of the present model might be the development of combinatorial and mutational assays to study the interactions between human TFs and their natural target promoters, which are not easily undertaken in tissue culture cells because of the variability in transfection efficiency, especially when multiple constructs are used. Thus, this novel system could be used to determine *in vivo* the genetic nature of the human mutant forms of these TFs, setting up a powerful tool to unravel the molecular genetic mechanisms that lead to CHDs. Developmental Dynamics 241:190–199, 2012. © 2011 Wiley Periodicals, Inc.

**Key findings:**

- The mammalian transcription factors GATA4, Nkx2.5 and TBX5 are able to transactivate the *Nppa* and *Myh6* human enhancers in *Drosophila* embryos.
- The endogenous *Drosophila* homologs of GATA4, Nkx2.5 and TBX5 are not transactivated by GATA4, Nkx2.5 or TBX5, ruling out a possible endogenous regulatory circuit.
- Overexpression in the mesoderm of GATA4, Nkx2.5 or TBX5 is lethal and overexpression in eyes and wings causes reduced organs.

## INTRODUCTION

The core network of transcription factors (TFs) and the main events that lead to heart formation have been conserved in evolution. The cardiac TFs GATA4, NKX2.5, and TBX5 are central to human heart development (Clark et al.,^2006^; Olson,^2006^; Srivastava,^2006^; Nemer,^2008^). Mutations in these genes significantly perturb heart development resulting in Congenital Heart Diseases (CHDs). In *Drosophila*, mutations in the homologues of these genes, *tinman* (*tin*; the fly *NKX2.5* homologue), *pannier* (*pnr*; the fly *GATA4* homologue), *Dorsocross1-3* (*Doc1-3*; most closely related to *Tbx6*), and *midline* (*mid*; the fly *TBX20* homologue), affect the formation of the cardiac tube (reviewed in Reim and Frasch,^2010^). Mutations in the two cardiac TFs GATA4 and NKX2.5 impair their functions leading to CHDs (Schott et al.,^1998^; Garg et al.,^2003^). In humans, *TBX5* is essential for heart and forelimb development and mutations in this gene lead to a developmental disorder called Holt-Oram syndrome (Basson et al.,^1997^; Li et al.,^1997^; Mori and Bruneau,^2004^). Mutations in TBX5 significantly alter its DNA-binding, protein–protein interaction and transcriptional activities (Ghosh et al.,^2001^; Hiroi et al.,^2001^; Fan et al.,^2003^; Garg et al.,^2003^). TBX5, alone or in combination with NKX2.5 and GATA4, regulates the transcription of *NPPA, MYH6* and *CX40*, genes that are important for cardiomyocyte differentiation (Bruneau et al.,^2001^; Hiroi et al.,^2001^; Ching et al.,^2005^). Functional interactions between TBX5 and GATA4 are important for heart development and compound heterozygous mice show significant down-regulation of *Myh6* (Maitra et al.,^2009^), which encodes a sarcomeric protein associated with cardiomyopathy and other congenital heart disorders (Carniel et al.,^2005^; Ching et al.,^2005^). Cardiac TFs Tbx5 and Gata4 in combination with Baf60c or Mef2c can also drive ectopic differentiation of mouse mesoderm or fibroblast cells into beating cardiac myocytes (Takeuchi and Bruneau,^2009^; Ieda et al.,^2010^), thereby suggesting the functional importance of these TFs and also their potential for future regenerative medicine.

Although functional cooperation between these cardiac TFs has been well documented, to date there are very few direct targets characterized. Two well-characterized targets are *NPPA* and *MYH6. NPPA* is a downstream target of GATA4 (Durocher et al.,^1997^; Lee et al.,^1998^), NKX2.5 (Durocher et al.,^1996^), and TBX5 (Hiroi et al.,^2001^). Mutations in either *NKX2.5* or *TBX5* severely reduce the transcription of *NPPA* (Zhu et al.,^2000^; Ghosh et al.,^2001^; Hiroi et al.,^2001^; Fan et al.,^2003^), whereas the transcription of *MYH6* is impaired by *TBX5* or *GATA4* mutations (Garg et al.,^2003^; Ching et al.,^2005^). The enhancers of these genes are invaluable tools for understanding the molecular mechanisms of heart development and disorders. Most commonly, these studies were conducted in *in vitro* tissue culture cells following transfection of the relevant constructs. Mutations in these TFs are pleiotropic and show different degrees of penetrance. Thus, their *in vivo* study in vertebrates is a difficult task.

It is known that regulatory regions identified in one organism can function in a heterologous organism (Awgulewitsch and Jacobs,^1992^; Malicki et al.,^1992^; Frasch et al.,^1995^; Pöpperl et al.,^1995^; Haerry and Gehring,^1996^,^1997^; Keegan et al.,^1997^; Xu et al.,^1999^; Brugger et al.,^2004^). For example, the autoregulatory elements of *Drosophila deformed* and of its mammalian homologue *Hoxb-4* are functionally very conserved as they are able to function in each heterologous organism (the fly homologue in mice and the human one in flies) (Awgulewitsch and Jacobs,^1992^; Malicki et al.,^1992^); the r4 enhancer of mouse *Hoxb-1* functions as an auto-regulatory element in *Drosophila* embryos (Pöpperl et al.,^1995^); the eye-specific enhancer of *Drosophila eyeless* directs eye- and CNS-specific expression in transgenic mice and the mouse *Pax6* P1 upstream region directs expression in *Drosophila* photoreceptors (Xu et al.,^1999^). All these examples highlight a high level of evolutionary conservation of the transcriptional machinery between insects and mammals.

In the present work, we show specific reporter gene up-regulation of two human enhancers by the mammalian TFs GATA4, Nkx2.5, and TBX5 in *Drosophila* embryos. *Drosophila* has been largely used as a model to study homologies of signaling and regulatory pathways that govern organogenesis in vertebrates and invertebrates. Rescue experiments where a *Drosophila* TF is replaced by its vertebrate homologue or where a vertebrate regulatory sequence is activated by a *Drosophila* TF have been performed. Nevertheless, it is worth mentioning that such assayed vertebrate regulatory sequences belong to TFs or to signaling molecules at the top of the pyramid of cues that lead to organogenesis. On the contrary, this has rarely been shown for downstream effector genes, which are the actual “realizators” (Garcia Bellido, 1977) of organogenesis. Since regulatory regions identified in one organism might function in a heterologous organism, we decided to investigate if this could be true also for the regulatory regions of downstream effector genes involved in human cardiogenesis. In this work, we show that the mammalian TFs GATA4, Nkx2.5, and TBX5 are able to up-regulate their natural target enhancers *in vivo* in *Drosophila* embryos.

## RESULTS AND DISCUSSION

In order to determine whether human enhancers of cardiac realizator genes could be active in *Drosophila*, we first selected two human enhancers belonging to genes that are involved in a widely described human pathology, namely CHDs, the regulation of which is well known. The two enhancers belong to the *NPPA* and *MYH6* human genes. ANF, the product of *NPPA*, is one of the earliest markers of cardiac differentiation expressed in the forming ventricular and atrial chamber myocardium (Christoffels et al.,^2000^). The *NPPA* promoter has served as a model for studying the regulatory mechanisms of cardiac TFs. A number of cardiac TFs such as GATA4 (Durocher et al.,^1997^), NKX2.5 (Durocher et al.,^1996^) and TBX5 (Ghosh et al.,^2001^; Hiroi et al.,^2001^) have been shown to interact with an *NPPA* enhancer. *MYH6* is another important structural and functional gene mainly expressed in the atrium (Kurabayashi et al.,^1988^). In humans, mutations in this gene are associated with atrial septal defects (Ching et al.,^2005^) and cardiomyopathy (Carniel et al.,^2005^). The *MYH6* enhancer is regulated by the TFs GATA4 (Molkentin et al.,^1994^), TBX5 (Ching et al.,^2005^), and MEF2 (Ghosh et al.,^2009^).

**Fig. 1:**
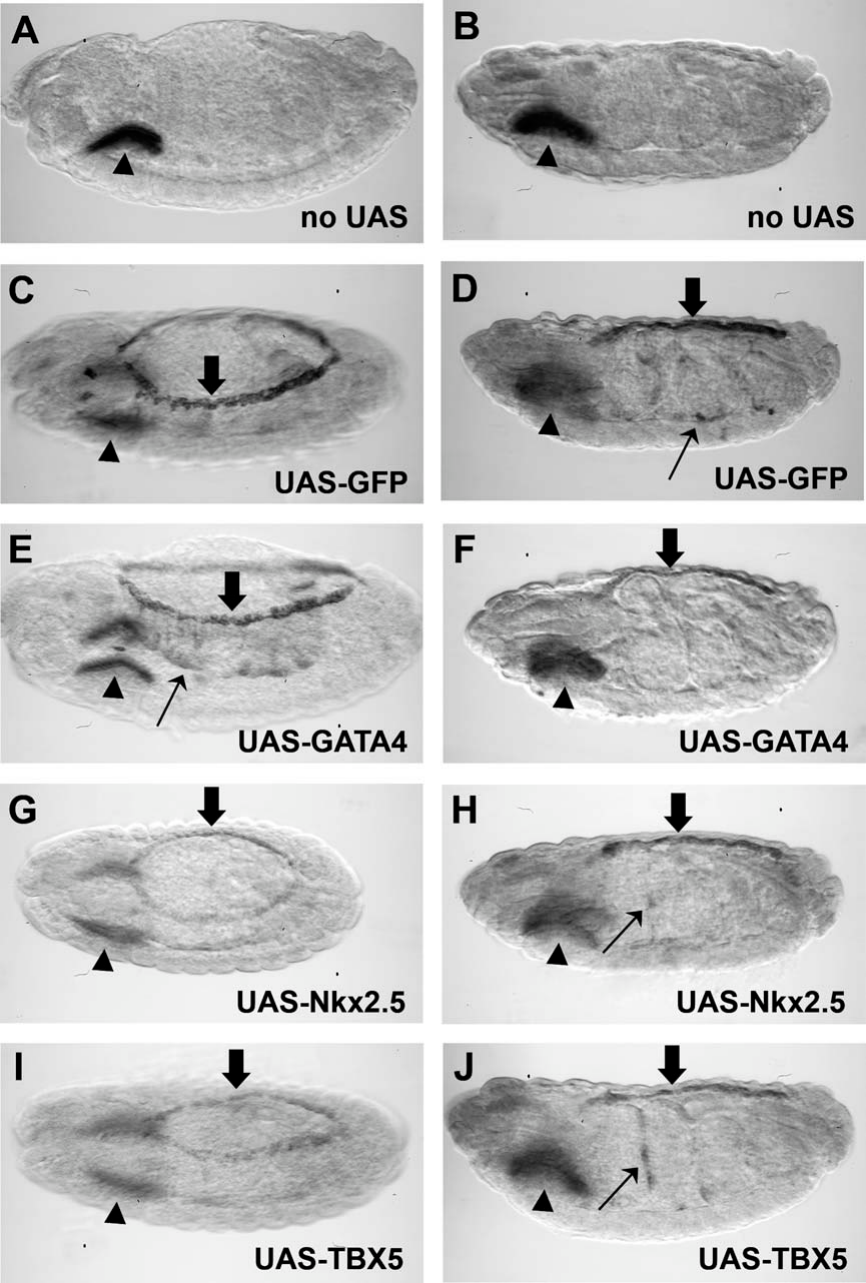
GATA4, Nkx2.5, and TBX5 transactivate the ANF300 enhancer in *Drosophila* embryos. Detection of *GFP* expression driven by the ANF300 enhancer by *in situ* hybridization using HRP staining. All embryos are shown with the anterior end to the left. Left column: Stage 3–14 embryos. Right column: Stage 15–16 embryos (dorsal side up). All embryos carry the *ANF-hs43-nGFP* reporter and the *Hand-GAL4* driver. In wild-type embryos (**A, B**), no *GFP* is detected. In embryos carrying also the *UAS-GFP* (**C, D**), *UAS-GATA4* (**E, F**), *UAS-Nkx2.5* (**G, H**), or *UAS-TBX5* (**I, J**) transgene, *GFP* is detected in the cardiac tube (block arrows) and in the visceral mesoderm (arrows). Staining with the sense *GFP* probe did not produce any signal except for salivary glands (arrowheads, data not shown).

**Fig. 2:**
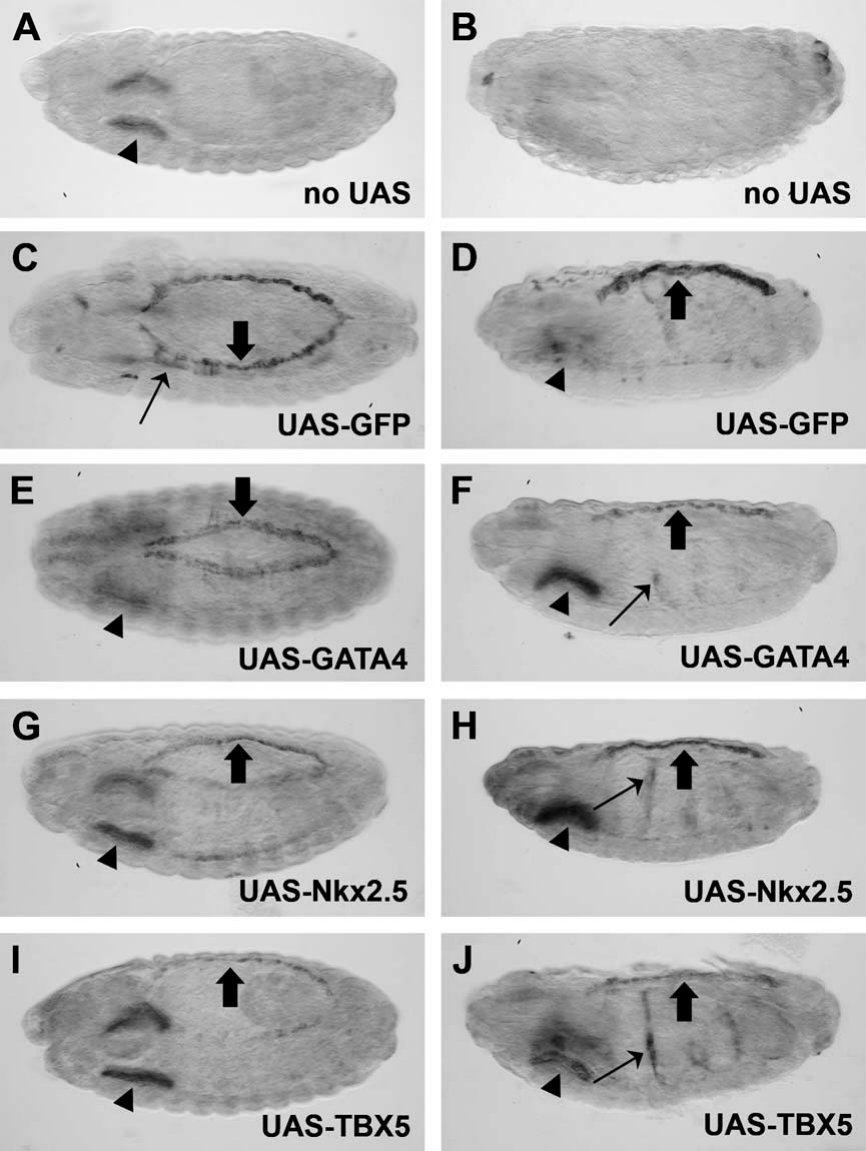
GATA4, Nkx2.5, and TBX5 transactivate the MYH6 enhancer in *Drosophila* embryos. Detection of GFP expression driven by the MYH6 enhancer by *in situ* hybridization using HRP staining. Left column: Stage-13–14 embryos. Right column: Stage-15–16 embryos (dorsal side up). All embryos carry the *MYH6-hs43-nGFP* reporter and the *Hand-GAL4* driver. In wild-type embryos (**A, B**), no *GFP* is detected. In embryos carrying also the *UAS-GFP* (**C, D**), *UAS-GATA4* (**E, F**), *UAS-Nkx2.5* (**G, H**), or *UAS-TBX5* (**I, J**) transgene, *GFP* is detected in the cardiac tube (block arrows) and in the visceral mesoderm (arrows). Staining with the sense *GFP* probe did not produce any signal except for salivary glands (arrowheads, data not shown).

In our study, we have used a 300-bp fragment immediately upstream of the *NPPA* promoter (called ANF300) because this enhancer fragment was shown to be activated *in vitro* by the three cardiac-specific TFs TBX5, NKX2.5, and GATA4 (Durocher et al.,^1997^; Bruneau et al.,^2001^; Ghosh et al.,^2001^). Similarly, the 4.5-Kb fragment of *MYH6* we used has also been shown to be activated *in vitro* by GATA 4 (Molkentin et al.,^1994^), by TBX5 (Ching et al.,^2005^), and by TBX5 in cooperation with MEF2C (Ghosh et al.,^2009^). We cloned the ANF300 and MYH6 enhancers in the pH-Stinger vector (Barolo et al.,^2000^) upstream of the hs43 promoter directing nGFP expression. No nGFP expression was detected in transgenic embryos by *in situ* hybridization (Figs. 1A, B, 2A, B). This indicates that wild-type levels of *Drosophila* TFs are unable to up-regulate these two enhancers either because the levels of GFP expression driven by the endogenous TFs are too low to be detected or because the endogenous TFs are busy taking care of their normal targets (a sort of threshold effect). By contrast, the over-expression of *GATA4, Nkx2.5*, or *TBX5* in embryos using the UAS/GAL4 system (Brand and Perrimon,^1993^) specifically activated *nGFP* in the same way through both the ANF300 (Fig. 1) and MYH6 (Fig. 2) enhancers. When the three TFs are over-expressed through the *Hand-GAL4* driver (Albrecht et al.,^2006^; Sellin et al.,^2006^), *GFP* expression is detected in the cardiac tube and in the visceral mesoderm (Figs. 1E–J, 2E–J), in the same domains as *UAS-GFP* expression (Figs. 1C,D, 2C,D). This indicates that the three human TFs are able to up-regulate their human targets in *Drosophila* and that *MYH6* is a putative target of *Nkx2.5*, which has not been reported before. No GFP expression was observed in embryos carrying only the reporter and the *Hand-GAL4* driver (Figs. 1A,B, 2A,B) or when over-expression was achieved in the Central Nervous System with the *elav-GAL4* driver (data not shown), which did not lead to lethality.

**Fig. 3:**
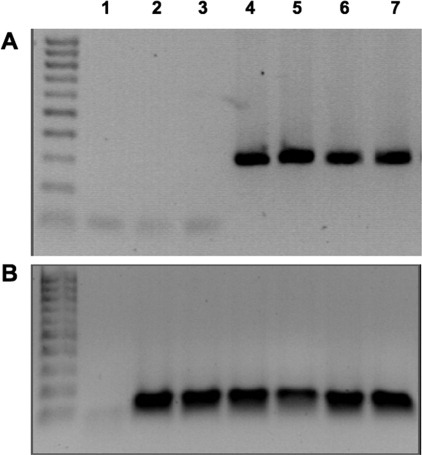
Detection of GFP by RT-PCR. RT-PCR was performed on the same embryos as those of Figure 1. **A:** RT-PCR detection of *GFP*. **B:** RT-PCR detection of the *rp49* housekeeping gene. 1, mock PCR; 2, *white*; 3, *Hand-GAL4; ANF300MYH6-hs43-nGFP*; 4, *Hand-GAL4; ANF300MYH6-hs43-nGFP* + *UAS-GATA4*; 5, *Hand-GAL4; ANF300MYH6-hs43-nGFP* + *UAS-Nkx2.5*; 6, *Hand-GAL4; ANF300MYH6-hs43-nGFP* + *UAS-TBX5*; 7, *Hand-GAL4; ANF300MYH6-hs43-nGFP* + *UAS-GFP*. A specific *GFP* band is detected only in *GATA4, Nkx2.5*, and *TBX5* overexpressing embryos (lanes 4–6) and in the positive control (lane 7).

We extracted mRNA from the embryos of the same genotypes as those used for the *in situ* hybridization shown in Figure 1. We reverse-transcribed it and used it as a template in PCR reactions amplified with GFP oligonucleotides. We did not observe any amplification product in embryos carrying only the reporter and the *Hand-GAL4* transgenes (Fig. 3A, lane 3), whereas we detected a specific band of the expected GFP size (Fig. 3A, lane 7) in the embryos that also carry the *UAS-GATA4, UAS-Nkx2.5*, or *UAS-TBX5* effector constructs (Fig. 3A, lanes 4–6). The *rp49* housekeeping control indicates that cDNA is equally present in all samples (Fig. 3B). The same RT-PCR experiment has been done for the MYH6 enhancer and gave the same results (data not shown). Thus, *GFP* is indeed up-regulated by the three TFs considered.

The *Drosophila* system we propose allows analyzing the functions of human TFs *in vivo. In vitro*, not all cell types are transfected with the same efficiency and, in some instances, overexpression of the TFs causes spurious activity. Using *Drosophila* transgenics as a model overrides variability, for example due to transfection efficiency, because in transgenic flies all the cells in a tissue carry both the reporter and the effector constructs, which are up-regulated all at the same level through the UAS/GAL4 system. Consequently, in this system the molecular interactions observed can be studied at a higher level of precision. In addition, a strong potential of the present model might come from the combinatorial and mutational assays of TFs on their target promoters, which are not easily undertaken in tissue culture cells because of the variability in transfection efficiency especially when multiple constructs are used.

**Fig. 4:**
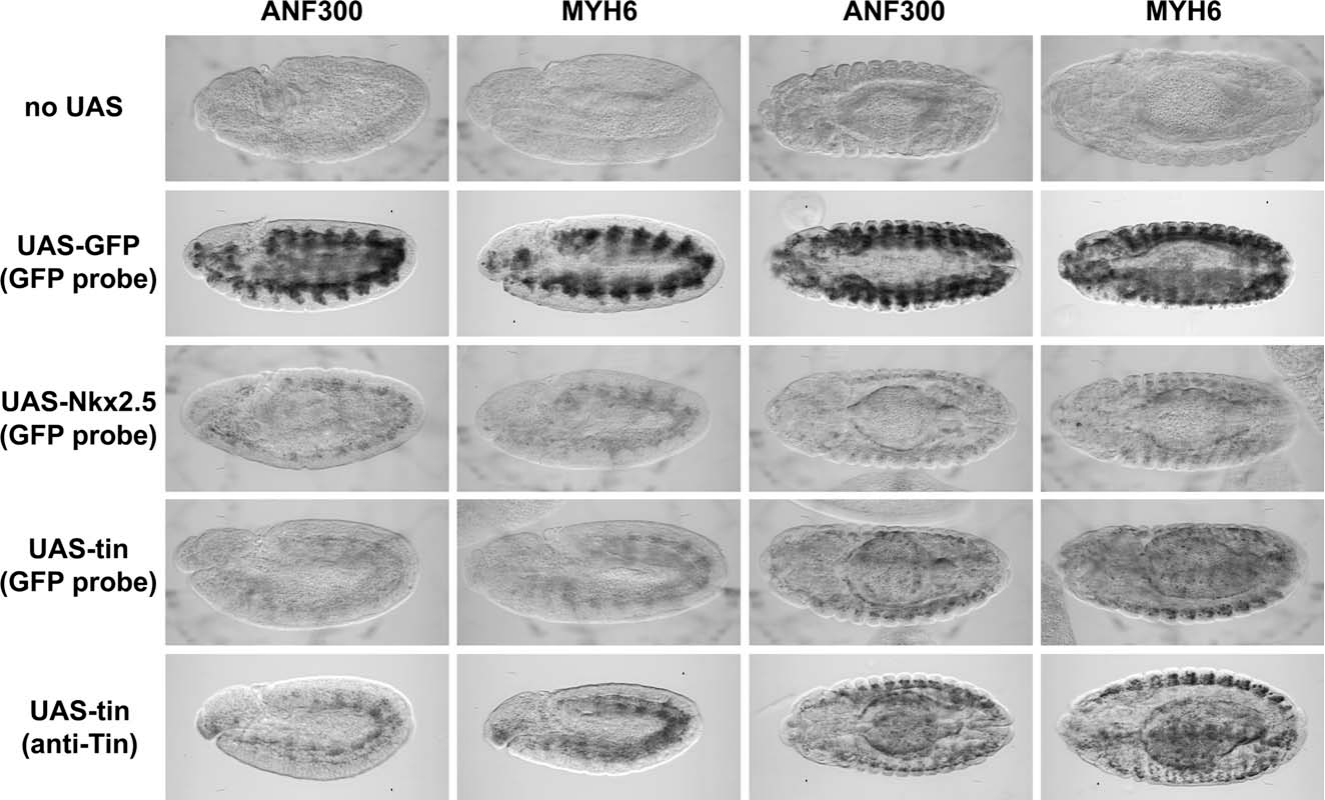
*tin* overexpression transactivates the ANF300 and MYH6 enhancers. Detection of *GFP* expression driven by the ANF300 and MYH6 enhancers upon *tin* overexpression by *in situ* hybridization. Left two columns: Germ band–extended embryos. Right two columns: Germ band–retracted embryos. Embryos carrying only the reporter and *GAL4* transgenes (top row) do not show *GFP* expression. Embryos overexpressing *GFP* (second row), *Nkx2.5* (third row), or *tin* (fourth row) through the *24B-GAL4* driver show *GFP* expression in the somatic and visceral (arrows) mesoderm. Overexpression of *tin* is detected by immunohistochemistry using anti-Tin antibodies (bottom row).

**Fig. 5:**
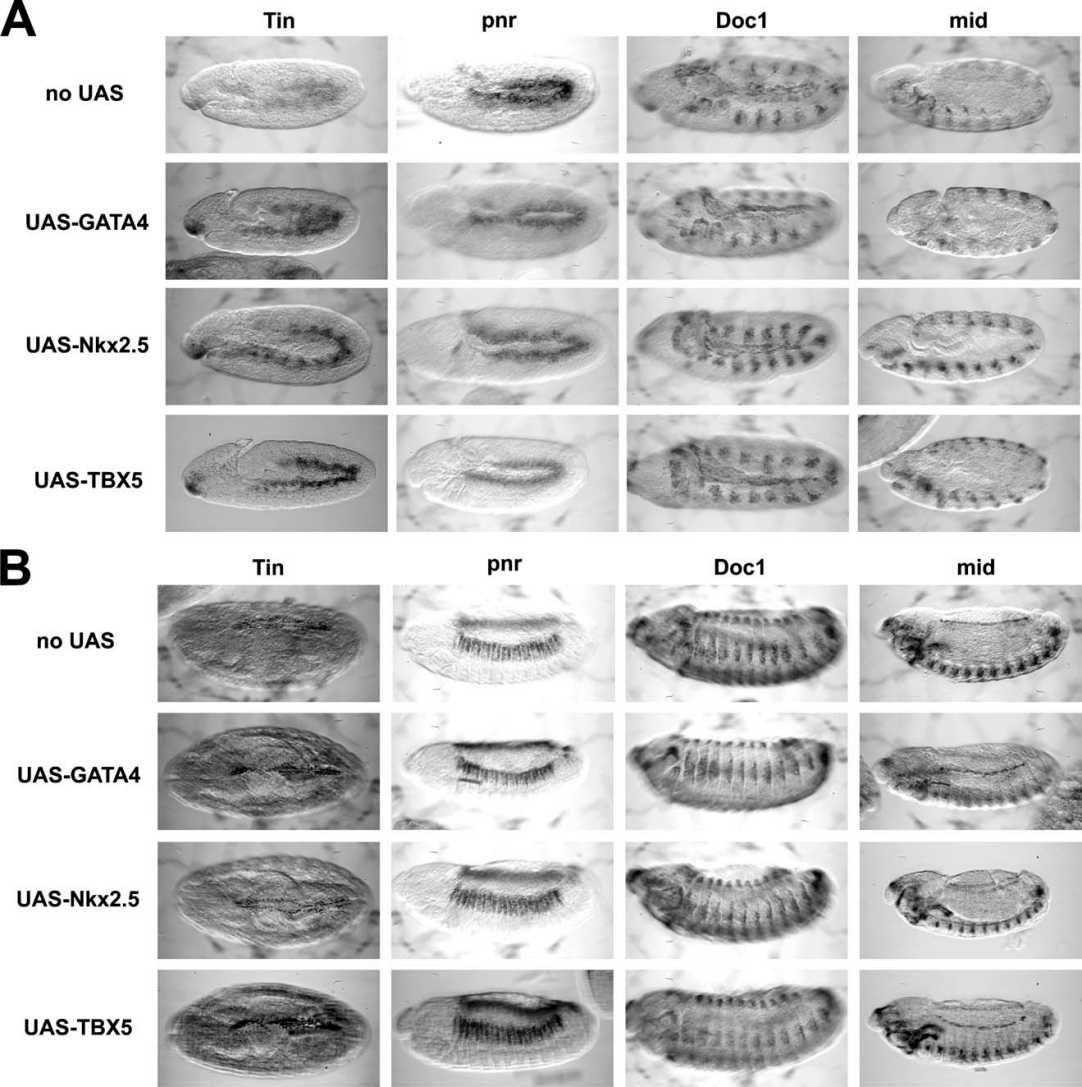
Endogenous *Drosophila* TFs are not mis-expressed by the over-expression of *GATA4, Nkx2.5*, or *TBX5*. Detection of *tin* protein by immunohistochemistry and of *pnr, Doc1*, and *mid* transcripts by *in situ* hybridization in control embryos (top) and in embryos overexpressing *GATA4, Nkx2.5*, or *TBX5* (see left) in the whole mesoderm through the *24B-GAL4* driver. **A:** Germ band–extended embryos. **B:** Germ band–retracted embryos. Each gene is expressed at normal levels in all embryos.

*pnr* and *tin* are the fly homologues of human *GATA4* and *NKX2.5*, respectively. The *Drosophila pnr, tin*, and *Tbx* genes are known to auto-regulate and to cross-regulate each other (Gajewski et al.,^2001^; Klinedinst and Bodmer,^2003^; Qian et al.,^2005^; Reim and Frasch,^2005^; Reim et al.,^2005^). Similarly, NKX2.5 regulates the expression of human *GATA4* (Riazi et al.,^2009^) and GATA4 also regulates transcription of *NKX2.5* through an upstream *NKX2.5* enhancer (Lien et al.,^1999^). So far, there is no report on an autoregulatory role for *NKX2.5* and *GATA4*. To rule out a possible endogenous regulatory circuit, we first determined whether *tin* (the fly homologue of *NKX2.5*) is able to up-regulate the ANF300 and MYH6 enhancers. Figure 4 shows the up-regulation of the two enhancers by overexpression of either *Nkx2.5* or *tin* in the whole mesoderm using the *24B-GAL4* driver, which directs expression in the whole mesoderm (Fig. 4; see Supp. Fig. S1, which is available online) (Zaffran et al.,^1997^). Thus, Tin has the same regulatory ability as its Nkx2.5 homologue in the regulation of the ANF300 and MYH6 enhancers. GATA4, Nkx2.5, and TBX5 could up-regulate the two enhancers through the indirect activation of their fly homologues. To rule out this possibility, we looked at the expression of the fly endogenous genes *pnr* (*GATA4* homologue), *tin* (*NKX2.5* homologue), *midline* (*TBX20* homologue), and *Doc1* (*TBX6* homologue; there is no true *Drosophila* homologue of *TBX5*) in embryos ectopically activating *GATA4, Nkx2.5*, or *TBX5*. By *in situ* hybridization (for *pnr, Doc1*, and *mid*) or immunohistochemistry (for *tin*), we did not see a change in expression of these endogenous fly genes in germ band–extended and in germ band–retracted embryos upon *GATA4, Nkx2.5*, or *TBX5* over-expression in the cardiac, somatic, and visceral mesoderm through the *24B-GAL4* driver (Fig. 5). This indicates that the regulation of the two tested enhancers by the three mammalian TFs is not indirect.

Several human and murine enhancers have been shown to be active in *Drosophila*. Most of the reporter constructs used in the regulatory studies on the evolutionary conservation of enhancers reported in the Introduction section (Awgulewitsch and Jacobs,^1992^; Malicki et al.,^1992^; Frasch et al.,^1995^; Pöpperl et al.,^1995^; Haerry and Gehring,^1996^,^1997^; Keegan et al.,^1997^; Xu et al.,^1999^; Brugger et al.,^2004^) utilized the TATA promoter *hs43* (the *Drosophila hsp70* promoter deleted of the Pelham heat-inducible box) that we used in our studies. This promoter is silent in *Drosophila*. It has been used in numerous studies on many types of *Drosophila* and mammalian TFs over many years (Qian et al.,^1991^,^1993^; Capovilla et al.,^1994^; Chan et al.,^1994^; Capovilla and Botas,^1998^; Hiromi and Gehring,^1987^; Wagner-Bernholz et al.,^1991^; Vachon et al.,^1992^; Gajewski et al.,^1997^; Ranganayakulu et al.,^1998^; Li et al.,^1999^; Ryoo et al.,^1999^; Han et al.,^2002^; Sellin et al.,^2006^; Ryan et al.,^2007^). In addition, several mammalian TFs have been shown to function in *Drosophila* as their fly homologues (Luo et al.,^1992^; Albagli et al.,^1996^; Ludlow et al.,^1996^; Deshpande et al.,^1997^; Leuzinger et al.,^1998^; Nagao et al.,^1998^; D'Souza et al.,^1999^; Fox et al.,^2010^). In most of these studies, the *Drosophila* or mammalian TFs were over-expressed through the UAS/GAL4 method we used. Thus, it is very likely that the GFP expression we observed comes from a direct interaction between the TFs over-expressed and the two human enhancers studied, which is supported by the results reported in Figure 5. To our knowledge, this is the first report of human enhancers being activated by human TFs in flies and it highlights the high evolutionary conservation of the regulatory functions of these molecules.

**Fig. 6:**
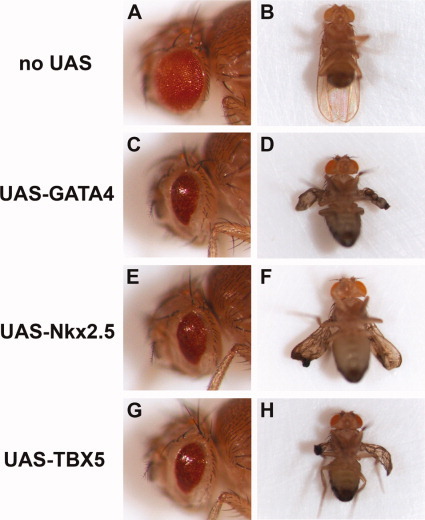
Developmental defects caused by *GAT4, Nkx2.5*, or *TBX5* over-expression in eyes and in wings. Reduced eyes and wings caused by the overexpression of *GATA4* (**C, D**), *Nkx2.5* (**E, F**) or *TBX5* (**G, H**) in the eye through the *GMR-GAL4* driver (C, E, G) or in the wing through the *vg-GAL4* driver (D, F, H). Top: Control flies carrying only the *GMR-GAL4* (**A**) or **vg-GAL4** (**B**) drivers. All flies were raised at 27°C, but phenotypic effects were visible also at 18°C (data not shown and Supp. Fig. S2).

We determined whether *GATA4, Nkx2.5*, or *TBX5* over-expression could cause developmental defects. To this aim, we over-expressed the three TFs in the whole mesoderm (through the *24B-GAL4* driver), in the eyes (through the *GMR-GAL4* driver), and in the wings (through the *vestigial-GAL4* driver). Over-expression of each of the three TF in the mesoderm is lethal. Over-expression in eyes and wings causes developmental defects, as both are very reduced (Fig. 6). For *GMR-GAL4*, the penetrance is complete at 28°C and at 18°C for all three TFs (Supp. Fig. S2). At 18°C, the overexpression of Nkx2.5 in the eye shows variable expressivity (data not shown). Finally, we observed that very similar phenotypes in both eyes and wings are elicited by the overexpression of *pnr* (with 100% penetrance at all temperatures), but not of *tin* (data not shown). This is consistent with the fact that *GATA4* is the true orthologue of *pnr* (Gajewski et al.,^1999^), whereas *Nkx2.5* is not able to functionally replace *tin* in all its developmental roles (Park et al.,^1998^; Ranganayakulu et al.,^1998^). The fact that overexpression of the three TFs leads to the same developmental defects suggests that they act in the same developmental pathway. Because the phenotypes elicited are viable and very easy to score, the over-expression of these TFs could be used to carry out modifier screens in order to identify novel interactors, as as it has been successfully done for other gene products (Fernandez-Funez et al.,^2000^; Bilen and Bonini,^2007^; Cukier et al.,^2008^; Jung et al.,^2010^).

To our knowledge, this is the first report of human TFs regulating their natural downstream effector targets in *Drosophila*. This system might be used to carry out structural/functional studies for combinatorial analyses of multiple TFs that would not be easily undertaken *in vitro*, because transfection efficiency is limiting when more than two factors are transfected. This assay may allow determining clearly the genetic functions of mutant forms of these TFs or of their targets involved in CHDs, to define unambiguously *in vivo* their inter-relationships and genetic features (e.g., dominance), which may help to understand the different phenotypes observed among CHDs patients.

## EXPERIMENTAL PROCEDURES

The GAL4 lines used are: *Hand*-*GAL4* (Albrecht et al., 2006; Sellin et al., 2006), P{GawB}*how*[24B] (*24B-GAL4*), *GMR-GAL4, elav-GAL4*, and *vestigial-GAL4*.

The ANF 300-bp enhancer fragment was amplified from the pGL3 ANF300 plasmid (Ghosh et al.,^2001^) using the oligonucleotides 5′-GGTCTAGACAGC GTCGAGGAGAAAGAAT-3′ (carrying a XbaI site) and 5′-TTGGATCCAGC TCTCCAAGCACGCAG-3′ (carrying a BamHI site). The 4.5-kb MYH6 enhancer was amplified from the pGL3 MYH6 4.5 plasmid (Ching et al.,^2005^) using the oligonucleotides 5′-CCTCTAGAGAAGCCGAGATCCT GACTCCAGACTCTTCT-3′ (carrying a XbaI site) and 5′-CCGGATCCCCT CTGTCTAAATTTGGAGTCCTTCCGG AG-3′ (carrying a BamHI site). Both enhancers were cloned in the XbaI and BamHI sites of pH-Stinger (Barolo et al.,^2000^) upstream of the minimal *hs43* promoter driving *nGFP* expression to generate the *ANF300-hs43-nGFP* and *MYH6-hs43-nGFP* reporters.

To generate the *UAS-GATA4* construct, the *GATA4* cDNA was excised from pcDNA3.1 Zeo(-) GATA4 (T. K. Ghosh, unpublished data) with EcoRI and cloned in the EcoRI site of pUAST (Brand and Perrimon,^1993^). The orientation was verified with BamHI to select a clone with the orientation appropriate for protein synthesis. The TBX5 cDNA was excised from pcDNA.1zeo(+) TBX5 (Ghosh et al.,^2001^) with XhoI and EcoRI and cloned in the NheI and BamHI sites of pBluescript KS+. From this construct, it was then excised using NotI and KpnI and cloned in the NotI and KpnI sites of pUAST to generate *UAS-TBX5. ANF300-hs43-nGFP*, and *MYH6-hs43-nGFP* transgenic flies were generated in the laboratory by standard methods. *UAS-GATA4* and *UAS-TBX5* transgenic flies were generated by Best Gene Inc. (Chino Hills, CA). *UAS-Nkx2.5* (Zaffran et al.,^2006^) flies and *UAS-tin* flies (Yin and Frasch,^1998^) were a gift of Manfred Frasch. At least two independent reporter and UAS lines were analyzed for each experiment.

The GAL4 lines used are: *Hand*-*GAL4* (Albrecht et al., 2006; Sellin et al., 2006), P{GawB}*how*[24B] (*24B-GAL4*), *GMR-GAL4, elav-GAL4*, and *vestigial-GAL4*.

### Crosses and Embryo Staining

Double-balanced *Hand-GAL4; ANF300-hs43-nGFP* and *Hand-GAL4; MYH6-hs43-nGFP* stocks were generated by standard procedures. Males of these stocks were crossed to *UAS-GFP* (Blooomington stock n. 1521), *UAS-GATA4, UAS-Nkx2.5*, or *UAS-TBX5* females at 27°C. Progeny embryos were dechorionated in 50% bleach, fixed in 4% formaldehyde, devitellinized in 100% methanol, and stored in 100% ethanol. All probes were made using the Riboprobe Combination System kit (Promega, Madison, WI) and the DIG RNA Labeling Mix (Roche, Indianapolis, IN). To make GFP probes, the GFP cDNA was excised from pStinger (Barolo et al.,^2000^) with EcoRI and XbaI and cloned in the EcoRI and XbaI sites of pGEM. *GFP* sense and antisense probes were made by linearizing with HindIII and transcribing with SP6 or by linearizing with EcoRI and transcribing with T7, respectively. The *Doc2, mid*, and *pnr* antisense probes were made as follows: the *Doc2* cDNA cloned in pNB40 (Reim et al.,^2003^) was linearized with SmaI and transcribed with T7, the *mid* cDNA plasmid cloned in pFLC (Reim et al.,^2005^) was linearized with XhoI and transcribed with T3, and the *pnr* cDNA plasmid (Ramain et al.,^1993^) cloned in pBluescript was linearized with HindIII and transcribed with T3. Embryos were stained using the TSA Plus Biotin System (Perkin Elmer, Waltham, MA) with minor modifications available upon request. Immunohistochemistry was carried out as previously described (Capovilla et al.,^2001^) with anti-Tin antibodies (Yin et al.,^1997^) at a 1:1,000 dilution.

### RT PCR

Total RNA was extracted from 30 μl of dechorionated 12–24-hr embryos using PureZOL RNA Isolation Reagent (BioRad, Hercules, CA) following instructions. cDNA was produced from 1 μg of total RNA digested with DNaseI using the iScript cDNA Synthesis kit (Bio-Rad) according to instructions. PCR was carried out using the following primers: GFP-For (5′-TGACCCTGAACTT CATCTG-3′), GFP-Rev (5′-GCTGTTGT AGTTGTACTC-3′), RP49-For (5′-TAT GCTAAGCTGTCGCAC-3′), and RP49-Rev (5′-ATCCGTAACCGATGTTGG-3′) using DreamTaq DNA Polymerase (Fermentas Life Sciences, Glen Burnie, MD).

## Supplementary material

Supp. Fig. S1. Pattern of GFP expression driven by the 24B-GAL4 driver. Detection of GFP expression by in situ hybridization in embryos carrying the 24B-GAL4 driver and the UAS-GFP transgene. A, B: Lateral (A) and dorsal (B) view of a germ band–extended embryo. C, D: Lateral (C) and dorsal (D) view of a germ band–retracted embryo. GFP is expressed in the whole mesoderm.

Supp. Fig. S2. The eye phenotype caused by GATA4, Nkx2.5, TBX5, or pnr overexpression is 100% penetrant. Low-magnification images of flies overexpressing GATA4 (A), Nkx2.5 (B), TBX5 (C), or pnr (D) through the GMR-GAL4 driver at 18°C. All flies show narrow eyes.

## References

[b1] Albagli O, Klaes A, Ferreira E, Leprince D, Klämbt C (1996). Function of ets genes is conserved between vertebrates and Drosophila. Mech Dev.

[b2] Albrecht S, Wang S, Holz A, Bergter A, Paululat A (2006). The ADAM metalloprotease Kuzbanian is crucial for proper heart formation in *Drosophila melanogaster*. Mech Dev.

[b3] Awgulewitsch A, Jacobs D (1992). Deformed autoregulatory element from Drosophila functions in a conserved manner in transgenic mice. Nature.

[b4] Barolo S, Carver L, Posakony J (2000). GFP and beta-galactosidase transformation vectors for promoter/enhancer analysis in Drosophila. Biotechniques.

[b5] Basson C, Bachinsky D, Lin R, Levi T, Elkins J, Soults J, Grayzel D, Kroumpouzou E, Traill T, Leblanc-Straceski J, Renault B, Kucherlapati R, Seidman J, Seidman C (1997). Mutations in human TBX5 [corrected] cause limb and cardiac malformation in Holt-Oram syndrome. Nat Genet.

[b6] Bilen J, Bonini N (2007). Genome-wide screen for modifiers of ataxin-3 neurodegeneration in Drosophila. PLoS Genet.

[b7] Brand AH, Perrimon N (1993). Targeted gene expression as a means of altering cell fates and generating dominant phenotypes. Development (Cambridge, England).

[b8] Brugger SM, Merrill AE, Torres-Vazquez J, Wu N, Ting M-C, Cho JY-M, Dobias SL, Yi SE, Lyons K, Bell JR, Arora K, Warrior R, Maxson R (2004). A phylogenetically conserved cis-regulatory module in the Msx2 promoter is sufficient for BMP-dependent transcription in murine and Drosophila embryos. Development (Cambridge, England).

[b9] Bruneau B, Nemer G, Schmitt J, Charron F, Robitaille L, Caron S, Conner D, Gessler M, Nemer M, Seidman C, Seidman J (2001). A murine model of Holt-Oram syndrome defines roles of the T-box transcription factor Tbx5 in cardiogenesis and disease. Cell.

[b10] Capovilla M, Botas J (1998). Functional dominance among Hox genes: repression dominates activation in the regulation of Dpp. Development.

[b11] Capovilla M, Brandt M, Botas J (1994). Direct regulation of decapentaplegic by Ultrabithorax and its role in Drosophila midgut morphogenesis. Cell.

[b12] Capovilla M, Kambris Z, Botas J (2001). Direct regulation of the muscle-identity gene apterous by a Hox protein in the somatic mesoderm. Development.

[b13] Carniel E, Taylor MRG, Sinagra G, Di Lenarda A, Ku L, Fain PR, Boucek MM, Cavanaugh J, Miocic S, Slavov D, Graw SL, Feiger J, Zhu XZ, Dao D, Ferguson DA, Bristow MR, Mestroni L (2005). Alpha-myosin heavy chain: a sarcomeric gene associated with dilated and hypertrophic phenotypes of cardiomyopathy. Circulation.

[b14] Chan S, Jaffe L, Capovilla M, Botas J, Mann R (1994). The DNA binding specificity of Ultrabithorax is modulated by cooperative interactions with extradenticle, another homeoprotein. Cell.

[b15] Ching Y, Ghosh T, Cross S, Packham E, Honeyman L, Loughna S, Robinson T, Dearlove A, Ribas G, Bonser A, Thomas N, Scotter A, Caves L, Tyrrell G, Newbury-Ecob R, Munnich A, Bonnet D, Brook J (2005). Mutation in myosin heavy chain 6 causes atrial septal defect. Nat Genet.

[b16] Christoffels V, Habets P, Franco D, Campione M, de Jong F, Lamers W, Bao Z, Palmer S, Biben C, Harvey R, Moorman A (2000). Chamber formation and morphogenesis in the developing mammalian heart. Dev Biol.

[b17] Clark K, Yutzey K, Benson D (2006). Transcription factors and congenital heart defects. Annu Rev Physiol.

[b18] Cukier H, Perez A, Collins A, Zhou Z, Zoghbi H, Botas J (2008). Genetic modifiers of MeCP2 function in Drosophila. PLoS Genet.

[b19] D'Souza J, Cheah PY, Gros P, Chia W, Rodrigues V (1999). Functional complementation of the malvolio mutation in the taste pathway of Drosophila melanogaster by the human natural resistance-associated macrophage protein 1 (Nramp-1). J Exp Biol.

[b20] Deshpande N, Chopra A, Rangarajan A, Shashidhara LS, Rodrigues V, Krishna S (1997). The human transcription enhancer factor-1, TEF-1, can substitute for Drosophila scalloped during wingblade development. J Biol Chem.

[b21] Durocher D, Chen C, Ardati A, Schwartz R, Nemer M (1996). The atrial natriuretic factor promoter is a downstream target for Nkx-2.5 in the myocardium. Mol Cell Biol.

[b22] Durocher D, Charron F, Warren R, Schwartz R, Nemer M (1997). The cardiac transcription factors Nkx2–5 and GATA-4 are mutual cofactors. Embo J.

[b23] Fan C, Liu M, Wang Q (2003). Functional analysis of TBX5 missense mutations associated with Holt-Oram syndrome. J Biol Chem.

[b24] Fernandez-Funez P, Nino-Rosales M, de Gouyon B, She W, Luchak J, Martinez P, Turiegano E, Benito J, Capovilla M, Skinner P, McCall A, Canal I, Orr H, Zoghbi H, Botas J (2000). Identification of genes that modify ataxin-1-induced neurodegeneration. Nature.

[b25] Fox RM, Hanlon CD, Andrew DJ (2010). The CrebA/Creb3-like transcription factors are major and direct regulators of secretory capacity. J Cell Biol.

[b26] Frasch M, Chen X, Lufkin T (1995). Evolutionary-conserved enhancers direct region-specific expression of the murine Hoxa-1 and Hoxa-2 loci in both mice and Drosophila. Development.

[b27] Gajewski K, Kim Y, Lee Y, Olson E, Schulz R (1997). D-mef2 is a target for Tinman activation during Drosophila heart development. Embo J.

[b28] Gajewski K, Fossett N, Molkentin J, Schulz R (1999). The zinc finger proteins Pannier and GATA4 function as cardiogenic factors in Drosophila. Development.

[b29] Gajewski K, Zhang Q, Choi C, Fossett N, Dang A, Kim Y, Kim Y, Schulz R (2001). Pannier is a transcriptional target and partner of Tinman during Drosophila cardiogenesis. Dev Biol.

[b30] Garcia-Bellido A (1977). Homeotic and atavic mutations in insects. Am Zool.

[b31] Garg V, Kathiriya I, Barnes R, Schluterman M, King I, Butler C, Rothrock C, Eapen R, Hirayama-Yamada K, Joo K, Matsuoka R, Cohen J, Srivastava D (2003). GATA4 mutations cause human congenital heart defects and reveal an interaction with TBX5. Nature.

[b32] Ghosh T, Packham E, Bonser A, Robinson T, Cross S, Brook J (2001). Characterization of the TBX5 binding site and analysis of mutations that cause Holt-Oram syndrome. Hum Mol Genet.

[b33] Ghosh TK, Song FF, Packham EA, Buxton S, Robinson TE, Ronksley J, Self T, Bonser AJ, Brook JD (2009). Physical interaction between TBX5 and MEF2C is required for early heart development. Mol Cell Biol.

[b34] Haerry T, Gehring W (1996). Intron of the mouse Hoxa-7 gene contains conserved homeodomain binding sites that can function as an enhancer element in Drosophila. Proc Natl Acad Sci USA.

[b35] Haerry T, Gehring W (1997). A conserved cluster of homeodomain binding sites in the mouse Hoxa-4 intron functions in Drosophila embryos as an enhancer that is directly regulated by Ultrabithorax. Dev Biol.

[b36] Han Z, Fujioka M, Su M, Liu M, Jaynes JB, Bodmer R (2002). Transcriptional integration of competence modulated by mutual repression generates cell-type specificity within the cardiogenic mesoderm. Dev Biol.

[b37] Hiroi Y, Kudoh S, Monzen K, Ikeda Y, Yazaki Y, Nagai R, Komuro I (2001). Tbx5 associates with Nkx2–5 and synergistically promotes cardiomyocyte differentiation. Nat Genet.

[b38] Hiromi Y, Gehring WJ (1987). Regulation and function of the Drosophila segmentation gene fushi tarazu. Cell.

[b39] Ieda M, Fu J-D, Delgado-Olguin P, Vedantham V, Hayashi Y, Bruneau BG, Srivastava D (2010). Direct reprogramming of fibroblasts into functional cardiomyocytes by defined factors. Cell.

[b40] Jung J, van Jaarsweld MTM, Shieh S-Y, Xu K, Bonini NM (2010). Defining genetic factors that modulate intergenerational CAG repeat instability in *Drosophila melanogaster*. Genetics.

[b41] Keegan LP, Haerry TE, Crotty DA, Packer AI, Wolgemuth DJ, Gehring WJ (1997). A sequence conserved in vertebrate Hox gene introns functions as an enhancer regulated by posterior homeotic genes in Drosophila imaginal discs. Mech Dev.

[b42] Klinedinst S, Bodmer R (2003). Gata factor Pannier is required to establish competence for heart progenitor formation. Development.

[b43] Kurabayashi M, Tsuchimochi H, Komuro I, Takaku F, Yazaki Y (1988). Molecular cloning and characterization of human cardiac alpha- and beta-form myosin heavy chain complementary DNA clones. Regulation of expression during development and pressure overload in human atrium. J Clin Invest.

[b44] Lee Y, Shioi T, Kasahara H, Jobe S, Wiese R, Markham B, Izumo S (1998). The cardiac tissue-restricted homeobox protein Csx/Nkx2.5 physically associates with the zinc finger protein GATA4 and cooperatively activates atrial natriuretic factor gene expression. Mol Cell Biol.

[b45] Leuzinger S, Hirth F, Gerlich D, Acampora D, Simeone A, Gehring WJ, Finkelstein R, Furukubo-Tokunaga K, Reichert H (1998). Equivalence of the fly orthodenticle gene and the human OTX genes in embryonic brain development of Drosophila. Development (Cambridge, England).

[b46] Li Q, Newbury-Ecob R, Terrett J, Wilson D, Curtis A, Yi C, Gebuhr T, Bullen P, Robson S, Strachan T, Bonnet D, Lyonnet S, Young I, Raeburn J, Buckler A, Law D, Brook J (1997). Holt-Oram syndrome is caused by mutations in TBX5, a member of the Brachyury (T) gene family. Nat Genet.

[b47] Li X, Veraksa A, McGinnis W (1999). A sequence motif distinct from Hox binding sites controls the specificity of a Hox response element. Development.

[b48] Lien CL, Wu C, Mercer B, Webb R, Richardson JA, Olson EN (1999). Control of early cardiac-specific transcription of Nkx2–5 by a GATA-dependent enhancer. Development (Cambridge, England).

[b49] Ludlow C, Choy R, Blochlinger K (1996). Functional analysis of Drosophila and mammalian cut proteins in files. Dev Biol.

[b50] Luo L, Tully T, White K (1992). Human amyloid precursor protein ameliorates behavioral deficit of flies deleted for Appl gene. Neuron.

[b51] Maitra M, Schluterman MK, Nichols HA, Richardson JA, Lo CW, Srivastava D, Garg V (2009). Interaction of Gata4 and Gata6 with Tbx5 is critical for normal cardiac development. Dev Biol.

[b52] Malicki J, Cianetti LC, Peschle C, McGinnis W (1992). A human HOX4B regulatory element provides head-specific expression in Drosophila embryos. Nature.

[b53] Molkentin J, Kalvakolanu D, Markham B (1994). Transcription factor GATA-4 regulates cardiac muscle-specific expression of the alpha-myosin heavy-chain gene. Mol Cell Biol.

[b54] Mori A, Bruneau B (2004). TBX5 mutations and congenital heart disease: Holt-Oram syndrome revealed. Curr Opin Cardiol.

[b55] Nagao T, Leuzinger S, Acampora D, Simeone A, Finkelstein R, Reichert H, Furukubo-Tokunaga K (1998). Developmental rescue of Drosophila cephalic defects by the human Otx genes. Proc Natl Acad Sci USA.

[b56] Nemer M (2008). Genetic insights into normal and abnormal heart development. Cardiovasc Pathol.

[b57] Olson E (2006). Gene regulatory networks in the evolution and development of the heart. Science.

[b58] Park M, Lewis C, Turbay D, Chung A, Chen J, Evans S, Breitbart R, Fishman M, Izumo S, Bodmer R (1998). Differential rescue of visceral and cardiac defects in Drosophila by vertebrate tinman-related genes. Proc Natl Acad Sci USA.

[b59] Pöpperl H, Bienz M, Studer M, Chan SK, Aparicio S, Brenner S, Mann RS, Krumlauf R (1995). Segmental expression of Hoxb-1 is controlled by a highly conserved autoregulatory loop dependent upon exd/pbx. Cell.

[b60] Qian L, Liu J, Bodmer R (2005). Neuromancer Tbx20-related genes (H15/midline) promote cell fate specification and morphogenesis of the Drosophila heart. Dev Biol.

[b61] Qian S, Capovilla M, Pirrotta V (1991). The bx region enhancer, a distant cis-control element of the Drosophila Ubx gene and its regulation by hunchback and other segmentation genes. EMBO J.

[b62] Qian S, Capovilla M, Pirrotta V (1993). Molecular mechanisms of pattern formation by the BRE enhancer of the Ubx gene. EMBO J.

[b63] Ramain P, Heitzler P, Haenlin M, Simpson P (1993). pannier, a negative regulator of achaete and scute in Drosophila, encodes a zinc finger protein with homology to the vertebrate transcription factor GATA-1. Development (Cambridge, England).

[b64] Ranganayakulu G, Elliott DA, Harvey RP, Olson EN (1998). Divergent roles for NK-2 class homeobox genes in cardiogenesis in flies and mice. Development (Cambridge, England).

[b65] Reim I, Frasch M (2005). The Dorsocross T-box genes are key components of the regulatory network controlling early cardiogenesis in Drosophila. Development.

[b66] Reim I, Frasch M (2010). Genetic and genomic dissection of cardiogenesis in the Drosophila model. Pediatr Cardiol.

[b67] Reim I, Lee H, Frasch M (2003). The T-box-encoding Dorsocross genes function in amnioserosa development and the patterning of the dorsolateral germ band downstream of Dpp. Development.

[b68] Reim I, Mohler J, Frasch M (2005). Tbx20-related genes, mid and H15, are required for tinman expression, proper patterning, and normal differentiation of cardioblasts in Drosophila. Mech Dev.

[b69] Riazi AM, Takeuchi JK, Hornberger LK, Zaidi SH, Amini F, Coles J, Bruneau BG, Van Arsdell GS (2009). NKX2–5 regulates the expression of beta-catenin and GATA4 in ventricular myocytes. PLoS ONE.

[b70] Ryan KM, Hendren JD, Helander LA, Cripps RM (2007). The NK homeodomain transcription factor Tinman is a direct activator of seven-up in the Drosophila dorsal vessel. Dev Biol.

[b71] Ryoo H, Marty T, Casares F, Affolter M, Mann R (1999). Regulation of Hox target genes by a DNA bound Homothorax/Hox/Extradenticle complex. Development.

[b72] Schott J, Benson D, Basson C, Pease W, Silberbach G, Moak J, Maron B, Seidman C, Seidman J (1998). Congenital heart disease caused by mutations in the transcription factor NKX2–5. Science.

[b73] Sellin J, Albrecht S, Kolsch V, Paululat A (2006). Dynamics of heart differentiation, visualized utilizing heart enhancer elements of the *Drosophila melanogaster* bHLH transcription factor Hand. Gene Expr Patterns.

[b74] Srivastava D (2006). Making or breaking the heart: from lineage determination to morphogenesis. Cell.

[b75] Takeuchi JK, Bruneau BG (2009). Directed transdifferentiation of mouse mesoderm to heart tissue by defined factors. Nature.

[b76] Vachon G, Cohen B, Pfeifle C, McGuffin M, Botas J, Cohen S (1992). Homeotic genes of the Bithorax complex repress limb development in the abdomen of the Drosophila embryo through the target gene Distal-less. Cell.

[b77] Wagner-Bernholz JT, Wilson C, Gibson G, Schuh R, Gehring WJ (1991). Identification of target genes of the homeotic gene Antennapedia by enhancer detection. Genes Dev.

[b78] Xu PX, Zhang X, Heaney S, Yoon A, Michelson AM, Maas RL (1999). Regulation of Pax6 expression is conserved between mice and flies. Development (Cambridge, England).

[b79] Yin Z, Frasch M (1998). Regulation and function of tinman during dorsal mesoderm induction and heart specification in Drosophila. Dev Genet.

[b80] Yin Z, Xu X, Frasch M (1997). Regulation of the twist target gene tinman by modular cis-regulatory elements during early mesoderm development. Development.

[b81] Zaffran S, Astier M, Gratecos D, Sémériva M (1997). The held out wings (how) Drosophila gene encodes a putative RNA-binding protein involved in the control of muscular and cardiac activity. Development (Cambridge, England).

[b82] Zaffran S, Reim I, Qian L, Lo P, Bodmer R, Frasch M (2006). Cardioblast-intrinsic Tinman activity controls proper diversification and differentiation of myocardial cells in Drosophila. Development.

[b83] Zhu W, Shiojima I, Hiroi Y, Zou Y, Akazawa H, Mizukami M, Toko H, Yazaki Y, Nagai R, Komuro I (2000). Functional analyses of three Csx/Nkx-2.5 mutations that cause human congenital heart disease. J Biol Chem.

