# Supplementary figures and images for: Transactivation in Drosophila of Human Enhancers by Human Transcription Factors Involved in Congenital Heart Diseases
Source: Dev Dyn. 2011 Oct 10;241(1):190–9. doi: 10.1002/dvdy.22763 (PMC3326377; doi:10.1002/dvdy.22763)

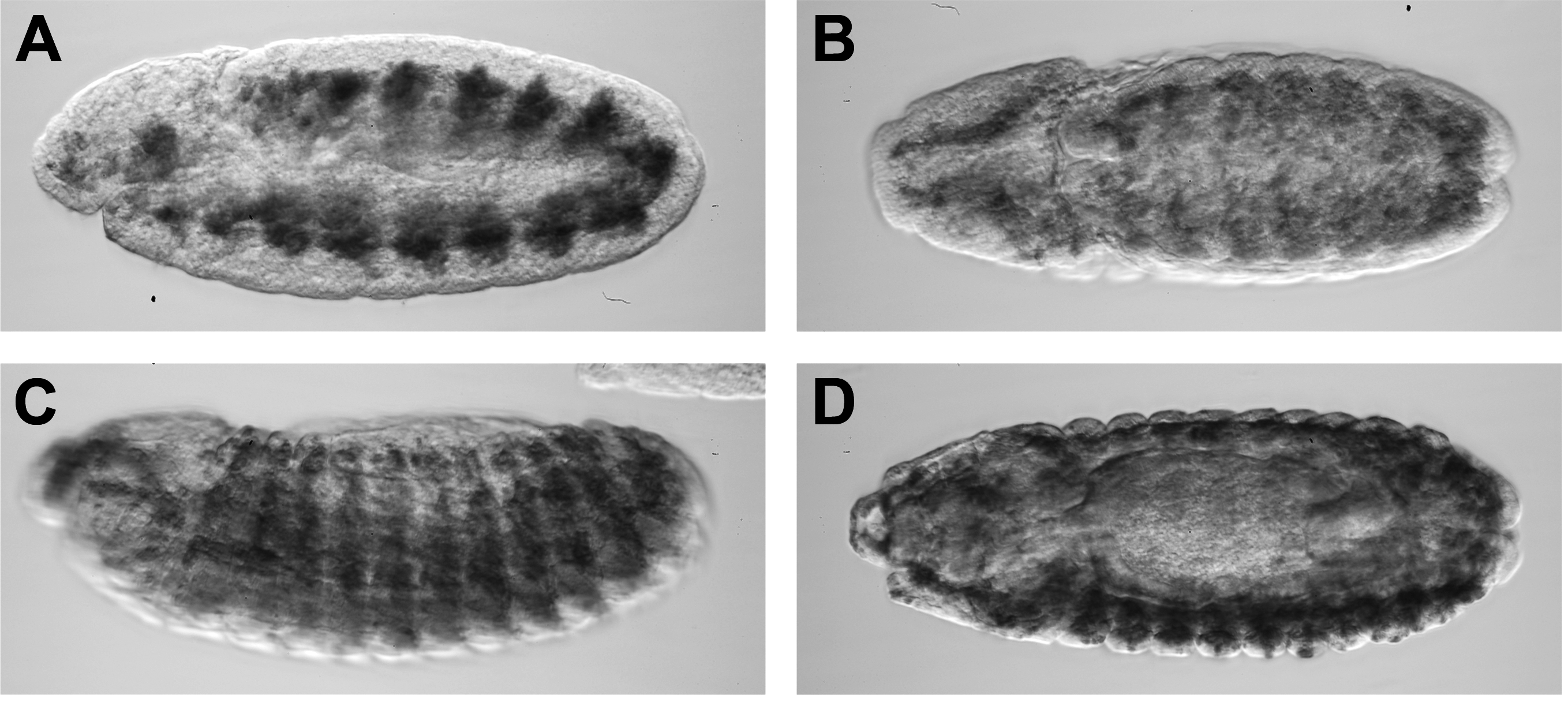

Supplement: Supplementary file 1 [file dvdy0241-0190-SD1.tif]

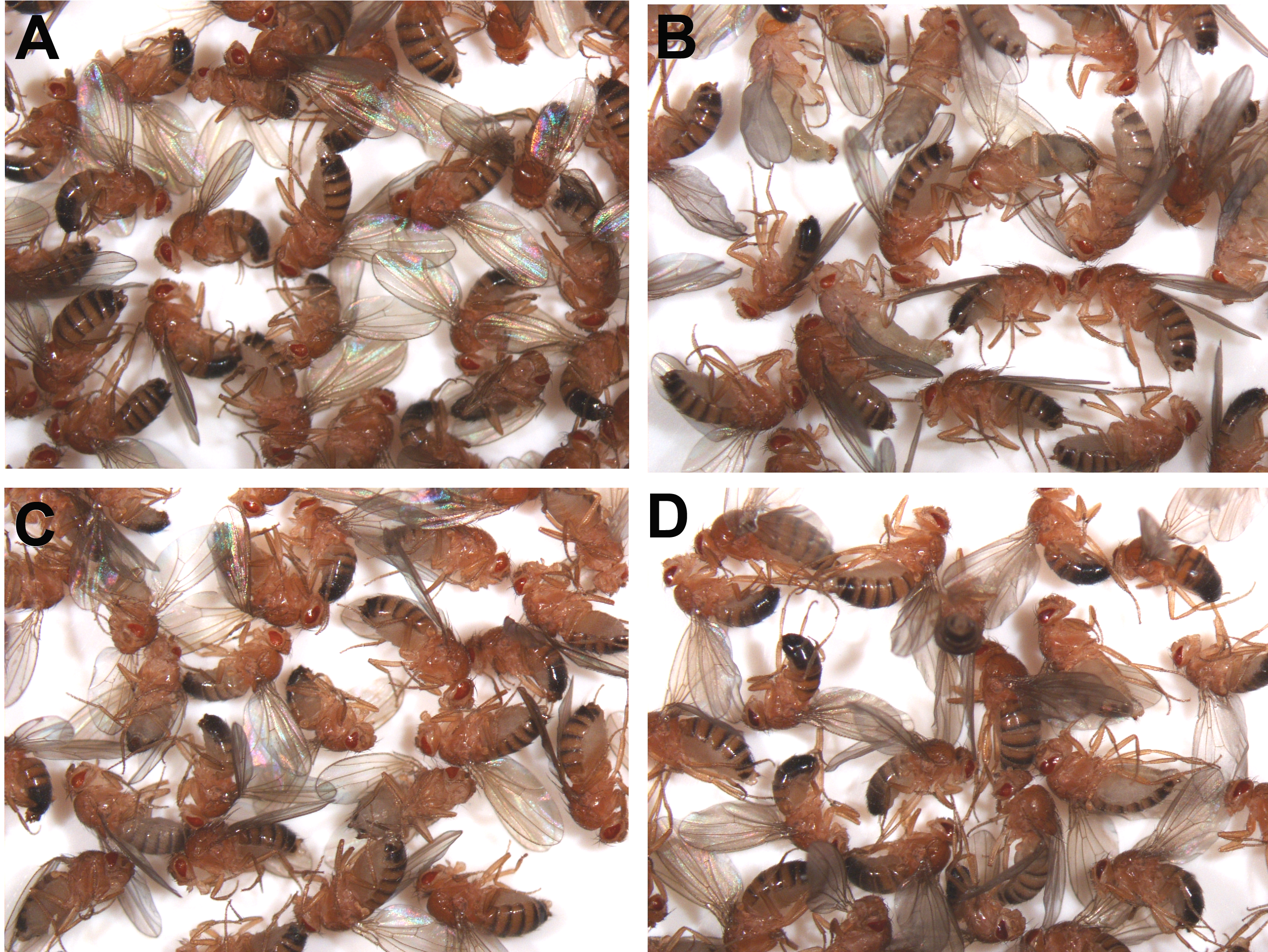

Supplement: Supplementary file 2 [file dvdy0241-0190-SD2.tif]
